# Urinary Complement Factor Ba: A New Tool for Early Detection of Acute Kidney Injury

**DOI:** 10.1016/j.ekir.2024.11.023

**Published:** 2024-11-23

**Authors:** Daan P.C. van Doorn, Augusto Cesar Soares dos Santos Junior, Sjoerd A.M.E.G. Timmermans

**Affiliations:** 1 Expertise Center for Immune-Mediated Kidney Diseases and Vasculitis, Maastricht University Medical Center, Maastricht, The Netherlands; 2 Department of Biochemistry, Cardiovascular Research Institute Maastricht, Maastricht University, Maastricht, The Netherlands; 3 Hospital das Clínicas, Ebserh Universidade Federal de Minas Gerais, Brazil; 4 Department of Internal Medicine, Faculdade Ciências Medicas, Minas Gerais, Brazil

See Clinical Research on Page 424

Acute kidney injury (AKI) is a pressing global health concern, with high rates of morbidity, including chronic and end-stage kidney disease, and mortality. AKI occurs in >20% of patients admitted to the hospital and is characterized by acute tubular injury, inflammation, and dysfunction of the kidneys’ vasculature.^1^ AKI is defined as a sudden loss of kidney function, typically identified through elevated serum creatinine and/or decreased urine output over a short period of time. Both markers, although widely accepted as markers of AKI, indicate a later stage of injury and thus, biomarkers that reflect AKI at the earliest possible stage of disease are needed to start appropriate treatment. Moreover, biomarkers may provide a better understanding of AKI’s mechanisms and pave the road to targeted treatment. Urine samples provide the potential to search for AKI-associated biomarkers related to, for example, inflammation. Leukocyte recruitment and inflammation in the kidneys result from ischemia-reperfusion injury and/or exposure to nephrotoxic agents in critically ill patients, at least in part, via complement activation.

The complement system consists of soluble and surface-bound proteins involved in the defense against pathogens and tissue homeostasis. Many of these proteins are so-called zymogens, that is, inactive precursor proteins that require cleavage to become activated, leading to opsonization, chemotaxis, and cytolytic immune responses. There are 3 main pathways of complement activation—the classical, lectin, and alternative pathway—each of which is triggered by different signals. Factor B, a central component of the alternative complement pathway, can bind to C3b (i.e., activated C3 component on surfaces) and cleaved into the amino-terminal Ba and carboxyl-terminal Bb fragments by factor D, forming the C3 convertase (i.e., C3bBb) that activates more C3 and the terminal complement pathway. Factor B has been linked to AKI in experimental ischemic AKI, with less severe disease in mice treated with factor B inhibition.^2^ The harmful effects of the alternative pathway of complement on tubular epithelial cells have been linked to ischemia-reperfusion injury.^3^ Urinary Ba, as a surrogate marker of factor B activation, was associated with severe AKI in a small cohort of critically ill children, either presenting with sepsis or not.^4^

In this issue of *Kidney International Reports*, Stenson *et al.*^5^ studied urinary Ba in 439 critically ill adult patients included in EARLYARF, either with AKI (i.e., stage ≤2^1^) or without AKI (Figure 1). Eligible patients with an expected intensive care unit length of stay > 24 hours and survival > 72 hours were enrolled. None of the patients had hematuria, rhabdomyolysis, or received chemotherapy for cancer. EARLYARF randomized critically ill patients at risk for AKI as based on urinary biomarkers for erythropoietin treatment, with no benefit as compared with placebo.^6^ The trial design, however, is suitable to investigate the dynamics of urinary Ba and its clinical correlates. Urine samples were collected at intensive care unit admission and again at 12 and 24 hours thereafter. Logistic regression analyses were used to assess the association between urinary Ba and AKI, with age and APACHE II score included as prespecified covariates.

**Figure 1:**
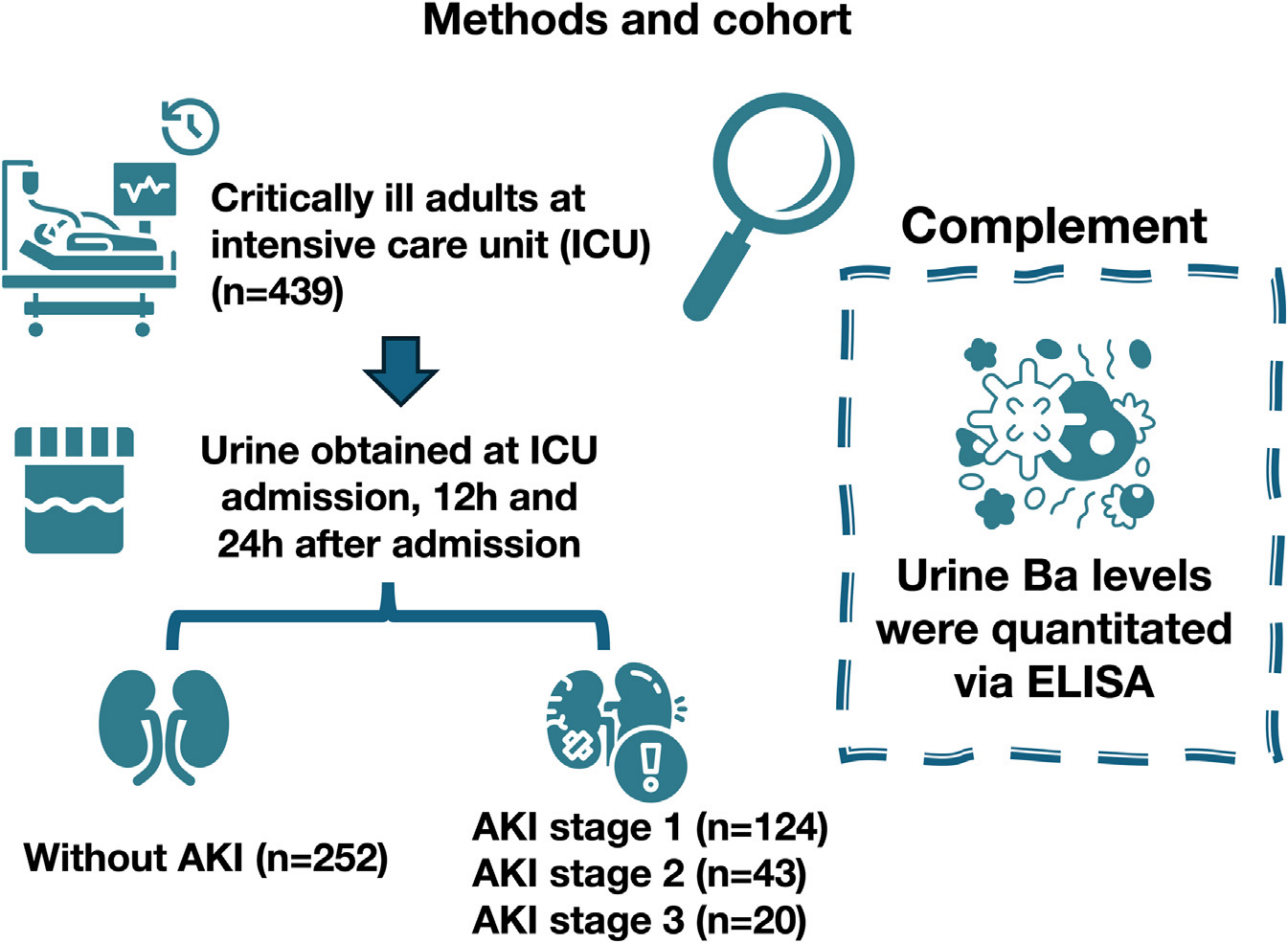
Study design: retrospective study assessing urine levels of complement factor Ba in 439 critically ill adult patients. Urine samples had been prospectively collected 15 years earlier and stored at −80 °C. Collections took place at intensive care unit admission and at 12 and 24 hours thereafter. Patients were divided into 2 groups—those with AKI and those without—and their urinary Ba levels were analyzed *post hoc*. AKI, acute kidney injury; ELISA, enzyme linked immunosorbent assay.

Urinary Ba levels were associated with AKI and correlated with AKI stage. In addition, urinary Ba levels were higher in patients with persistent AKI as compared with those who recovered kidney function. The authors suggest that urinary Ba may function as both a biomarker and mediator of AKI in critically ill patients and although captivating, their data should be interpreted with caution. First, knowledge about the kinetics of urinary biomarkers is warranted, given the kidneys’ complex processes that include glomerular filtration and tubular transport. Previous studies demonstrated that activation of complement via the alternative pathway enhances ischemic acute tubular injury,^2^^,^^3^ a common phenomenon in critically ill patients. Many such patients present with increased Ba levels in plasma because of systemic activation of the complement. The molecular mass of Ba is approximately 33 kDa; and thus, can be filtered through the glomerular filtration barrier into urine. Ba levels in plasma increase in patients with impaired kidney function,^7^ pointing to Ba handling by the kidneys. Urinary Bb, the larger fragment of factor B with a molecular mass of approximately 60 kDa, may therefore be more reliable as compared with Ba. Moreover, AKI in critically ill patients relates to ischemic acute tubular injury and is associated with proteinuria that, in addition to systemic activation of complement, may affect urinary Ba.^7^ Neither plasma samples to correct for circulating Ba levels nor data on proteinuria were available. Future studies on urinary Ba should therefore correct for circulating Ba levels and proteinuria before firm conclusions can be drawn. Second, measurement of complement split products is challenging because accurate and standardized sampling, processing, and storage is required to prevent *in vitro* complement activation.^8^ EARLYARF processed urine immediately and stored samples at −80 °C for approximately 15 years, with at least 1 thaw-freeze cycle. It is unknown whether a protease inhibitor was applied, making a comparison with other studies impossible. Despite these concerns, urinary Ba as a predictor of AKI and its course in critically ill patients is biologically plausible and thus, has potential clinical implications.

Ever since the introduction of eculizumab, the first approved anticomplement drug in humans, a wide selection of drugs is being developed and/or studied for various diseases, including cardiac surgery patients at risk for AKI (ARTEMIS; ClinicalTrials.gov ID: NCT05746559). ARTEMIS studies the effects of ravulizumab, an anti-C5 mAb, on major adverse kidney events, including AKI. C5 inhibition impaired leukocyte recruitment, cytolytic immune responses on the parenchyma, and attenuated AKI in experimental ischemic AKI.^9^ Of note, activation of C5 and the terminal complement pathway occurred late after ischemia as compared with C3 activation, and proximal complement inhibition may therefore be more effective. We look forward to future studies that assess the effects of various compounds that target the complement system at different levels of risk for AKI and persistent disease.

Knowledge about the dynamics of urinary complement split products, including Ba, may guide clinicians’ choice in mechanistic targets. The next step forward therefore involves the prospective evaluation of specificity, sensitivity, and predictive values of urinary complement split products to predict risk for AKI. Although additional validation is required, Stenson *et al.*^5^ underscore a potential role of the complement system in the development of AKI. The use of such biomarkers may be particularly beneficial for the identification of patients at the highest risk for AKI at the earliest possible stage of disease, and to differentiate patients who likely recover kidney function from those who may require kidney replacement therapy despite current treatment strategies. These insights could make it possible for future research into innovative treatments for patients who do not recover from AKI, an area still in need of advancement.

## Disclosure

SAMEGT participated in TMA expert meetings and received travel/speaker fees from Alexion Pharmaceutical Inc. (AstraZeneca). All the other authors declared no competing interests,
